# Low Cognitive Function Is Associated With Reduced Functional Fitness Among Community-Dwelling Older Adults

**DOI:** 10.1093/geroni/igaa057.512

**Published:** 2020-12-16

**Authors:** Michelle Gray, Joshua Gills, Jordan Glenn, Erica Madero, Aidan Hall, Nami Fuseya, Jennifer Vincenzo, Nick Bott

**Affiliations:** 1 University of Arkansas, Fayetteville, Arkansas, United States; 2 Neurotrack Technologies, Redwood City, United States; 3 Neurotrack Technologies, Redwood City, California, United States; 4 UAMS, Fayetteville, Arkansas, United States

## Abstract

Among older adults over 70, 22-30% report difficulty performing at least one activity of daily living (ADL). While the precipitants of ADL decline are multifactorial, over 50% of cognitively impaired adults require assistance with ADLs. The exact relationship between cognitive and functional decline remains unknown, but it is important to understand their relationship. Eighty-three older adults (80.9 + 5.4 years) enrolled in this study and completed functional fitness and cognitive assessments. Functional fitness assessments included: Short Physical Performance Battery (SPPB), 10-meter walk, dual-task, and power chair stand (average and peak). Cognition was assessed using the Montreal Cognitive Assessment (MoCA) and Visual Paired Comparison task (VPC). Categories of low cognitive function (LCF) and high cognitive function (HCF) were determined by VPC scores. SPPB was 10.2% greater among the HCF group. The HCF group walked 12.6% (0.16 m/s) faster than the LCF group. Dual-task (fast) performance was 13.2% faster among the HCF group. Additionally, when rising from a seated position during the average and peak power chair stand task, the HCF group moved 16.7% and 16.1% faster than the LCF group, respectively. MoCA scores were 2.8 points greater among the HCF group. Based on the current results, significant differences exist between cognitive groups suggesting a relationship between functional fitness and cognition. What remains unknown is the ability to influence functional fitness by changing cognition or vice versa. Future research is warranted to determine the relationship of change in either domain over time.

